# Dilated Blood and Lymphatic Microvessels, Angiogenesis, Increased Macrophages, and Adipocyte Hypertrophy in Lipedema Thigh Skin and Fat Tissue

**DOI:** 10.1155/2019/8747461

**Published:** 2019-03-03

**Authors:** Sara AL-Ghadban, Walter Cromer, Marisol Allen, Christopher Ussery, Michael Badowski, David Harris, Karen L. Herbst

**Affiliations:** ^1^Department of Medicine and TREAT Program, College of Medicine, University of Arizona, Tucson, Arizona, USA; ^2^Department of Medical Physiology, Texas A&M University Health Science Center, Temple, Texas, USA; ^3^Department of Immunobiology and Biorepository, College of Medicine, University of Arizona, Tucson, Arizona, USA

## Abstract

**Background and Aim:**

Lipedema is a common painful SAT disorder characterized by enlargement of fat primarily in the legs of women. Case reports of lipedema tissue samples demonstrate fluid and fibrosis in the interstitial matrix, increased macrophages, and adipocyte hypertrophy. The aims of this project are to investigate blood vasculature, immune cells, and structure of lipedema tissue in a cohort of women.

**Methods:**

Forty-nine participants, 19 controls and 30 with lipedema, were divided into groups based on body mass index (BMI): Non-Obese (BMI 20 to <30 kg/m^2^) and Obese (BMI 30 to <40 kg/m^2^). Histological sections from thigh skin and fat were stained with H&E. Adipocyte area and blood vessel size and number were quantified using ImageJ software. Markers for macrophages (CD68), mast cells (CD117), T cells (CD3), endothelial cells (CD31), blood (SMA), and lymphatic (D2-40 and Lyve-1) vessels were investigated by IHC and IF.

**Results:**

Non-Obese Lipedema adipocyte area was larger than Non-Obese Controls (*p*=0.005) and similar to Obese Lipedema and Obese Controls. Macrophage numbers were significantly increased in Non-Obese (*p* < 0.005) and Obese (*p* < 0.05) Lipedema skin and fat compared to Control groups. No differences in T lymphocytes or mast cells were observed when comparing Lipedema to Control in both groups. SMA staining revealed increased dermal vessels in Non-Obese Lipedema patients (*p* < 0.001) compared to Non-Obese Controls. Lyve-1 and D2-40 staining showed a significant increase in lymphatic vessel area but not in number or perimeter in Obese Lipedema participants (*p* < 0.05) compared to Controls (Obese and Non-Obese). Areas of angiogenesis were found in the fat in 30% of lipedema participants but not controls.

**Conclusion:**

Hypertrophic adipocytes, increased numbers of macrophages and blood vessels, and dilation of capillaries in thigh tissue of non-obese women with lipedema suggest inflammation, and angiogenesis occurs independent of obesity and demonstrates a role of altered vasculature in the manifestation of the disease.

## 1. Introduction

Lipedema is a common painful subcutaneous adipose tissue (SAT) disorder described in 1940 by Allen and Hines [1]. Lipedema develops during hormonal changes including pregnancy, childbirth, or menopause and may affect approximately 11% of adult women worldwide [2]. Lipedema is characterized by a disproportion of nodular, painful fat on the lower body compared to the trunk; arms are also affected in 80% of patients [2, 3]. Women with lipedema are often misdiagnosed with obesity or lymphedema. Importantly, lipedema fat is highly resistant to diet and exercise [4–7].

There are three stages of lipedema: Stage 1 with flat skin over pearl-sized nodules in a hypertrophic fat layer; Stage 2 with skin indentations over a hypertrophic fat structure of pearl-to-apple-size masses; and Stage 3 with pearl-sized nodules and much larger fat masses causing lobules of skin and fat to form particularly on the hips, thighs, and around the knees. Lymphedema, causing fluid accumulation in the limbs, may develop during any stage of lipedema especially Stage 3 [2], referred to as lipolymphedema. Flow in lymphatics in early stages of lipedema appears normal but can become impeded in later stages accompanied by microaneurysms and leak [7]. Furthermore, hypermobility, venous disease, and obstructive sleep apnea are common in women with lipedema, predominantly Stage 3 [2].

Histology of lipedema fat found in case reports demonstrates hypertrophy and hyperplasia of adipocytes associated with replicating mesenchymal stem cells [8], dilation of subdermal blood capillaries and perivascular cells, fibrosis of arterioles, and fibrosis and dilation of venules [9]. One report found increased numbers of blood vessels especially capillaries and prominent venules [10]. Large clusters of macrophages [11] and oil cysts [10, 12] have been reported.

This work investigates the histology of thigh skin and fat in women living with lipedema focusing on adipocyte size, immune cells infiltrating the tissue, and blood and lymphatic vessel size and number to gain insight into whether these tissue components might provide some insight into the pathophysiology of lipedema.

## 2. Materials and Methods

### 2.1. Participants

All participants provided written informed consent prior to participation. Protocols were reviewed and approved by the Human Research and Protection Program at the University of Arizona (Table 1). Participants were determined to have lipedema based on the criteria of Wold et al. [13]. Women with lipedema had symmetric hypertrophy of leg fat with pearl-sized nodules, tenderness to palpation, reported swelling and difficulty, and a disproportion of fat on the legs compared to the trunk that was difficult to lose by diet or exercise.

### 2.2. Biopsy

Punch biopsies (5 mm) were obtained from the anterolateral thigh. After injection of 1.5 ml (3.78 *μ* mole) lidocaine 2% (sc-362977Rx, TX, USA), skin and fat were removed and placed immediately into 10% formalin containers. The wound was closed with 2–4 absorbable sutures and covered in gauze and Tegaderm.

### 2.3. Slide Preparation

Formalin-fixed tissue samples were embedded in paraffin, and 5 *μ*m sections were cut for H&E staining, immunohistochemistry, and immunofluorescence.

#### 2.3.1. Adipocyte Size

All images were captured at 20x magnification (objective lens) using an EVOS XL Cell Imaging System. A total of 150 adipocytes were measured in 5 different fields per participant from H&E-stained histological sections. Analysis of adipocyte area was performed with ImageJ software (http://rsbweb.nih.gov/ij/). Adipocyte size greater than 100 *μ*m in diameter is considered hypertrophic [14].

#### 2.3.2. Immunohistochemistry

Tissue sections (skin and fat) were stained with markers for macrophages (CD68, DAKO/Agilent M0814, CA, USA), mast cells (CD117, BioCare CM5296C, CA, USA), T lymphocytes (CD3, Leica NCL-L-CD3-565, IL, USA), endothelial cells (CD31, DAKO/Agilent M0823, CA, USA), blood vessels (SMA, Thermo MS-113-P, MA, USA), and lymphatic vessels (D2-40, Biolegend SIG-3730, CA, USA), counterstained with hematoxylin; positive and negative controls were included (San Diego Pathologists Medical Group, CA, USA). Angiogenesis was assessed by Masson's trichrome stain (Sigma kit HT102A-1KT, MO, USA). All sections were coded and scored in a blinded manner by two independent investigators. The quantification of CD3, CD68, and CD117 positive cells was expressed as the average of total number of cells counted in 10 fields at 20x magnification (five fields in skin and five in fat).

#### 2.3.3. Immunofluorescence

Tissue sections were deparaffinized using standard xylene through ethanol (100%, 95%, 90%, 80%, and 70%) steps (15 min each). After de-paraffinization, the slides were placed into 0.01% sodium borohydride for 30 min for mild antigen retrieval. The slides were then blocked in 10% normal goat serum for 2 hours at room temperature and then incubated in primary antibody (Lyve-1, abcam, ab14917) 1 : 200 in 1% normal goat serum/0.01% triton X-100 in PBS overnight. The slides were then washed in PBS and incubated with secondary antibody (goat anti-rabbit AlexaFluor 488) 1 : 1000 for 2 hours at room temperature and then washed 3 times in PBS. The slides were mounted using Prolong Gold Antifade reagent with DAPI.

### 2.4. Blood Vessels

Blood vessels were counted at 20X magnification in 10 fields in SMA-stained sections. Arterioles, venules, and capillaries were counted in the dermis of skin including the epidermal/subpapillary (upper) and papillary (lower papillary dermis above or at the junction of the reticular dermis); capillaries were counted, and the diameter was measured in the hypodermal fat using ImageJ software.

### 2.5. Lymphatic Vessels

Examination of lymphatic vessels was limited to the dermal skin (papillary and reticular layers) as there were few lymphatic vessels within the hypodermal fat. Dermal tissue sections stained with Lyve-1 or D2-40 were imaged at 20X magnification (3 random fields of view per sample); vessel structures identified by endothelial staining were measured (maximum 10 and minimum 6 vessels per tissue). The luminal perimeter of the vessels in the tissue (determined by endothelial staining) was traced in ImageJ, and the perimeter, area, and aspect ratio were measured and averaged for each sample (Figure S1, Supplementary Materials for Lymphatic methodology). There were no differences within groups for vessel measurements from Lyve-1 and D2-40 stained tissues.

### 2.6. Statistical Analyses

GraphPad PRISM 5 (Version 5.03) was used for all statistical analyses. Results are displayed as minimum/maximum box-whisker plots where the median value is indicated by the solid horizontal line within each box. The Mann–Whitney test was used to determine the differences between the two groups of participants. One-way ANOVA followed by Tukey's post hoc test was used to analyze the differences between the four groups. Asterisks (^*∗*^) indicate statistical significance: ^*∗*^ *p* < 0.5; ^*∗∗*^ *p* < 0.01; ^*∗∗∗*^ *p* < 0.001.

## 3. Results

### 3.1. Demographics

Forty-nine participants, 19 non-lipedema controls and 30 with lipedema, were divided into two groups based on BMI (Table 1): Non-Obese (BMI 20 to <30 kg/m^2^) and Obese (BMI 30 to <40 kg/m^2^). Three lipedema participants had prediabetes and none had diabetes; none of the controls had prediabetes or diabetes.

### 3.2. Adipocyte Area

**Figure 1:**
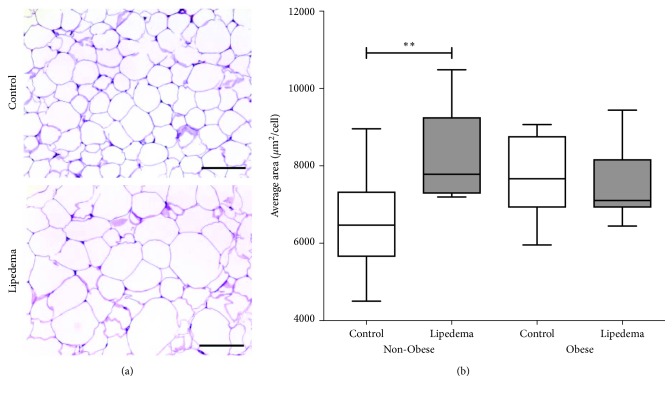
Adipocyte area measured in Non-Obese and Obese participants with and without lipedema (controls). (a) Representative H&E images showing heterogeneity and hypertrophy in cell size in adipocytes in leg fat of Non-Obese participants (scale bar = 200 *µ*m). (b) Box and whisker plot representing the average adipocyte area in Non-Obese (Control *n*=10; Lipedema *n*=10) and Obese groups (Control *n*=9; Lipedema *n*=11) (^*∗*^ *p* < 0.05; ^*∗∗*^ *p* < 0.01).

Heterogeneity in adipocyte cell size was observed in fat from all participants, ranging from 40 to 200 *μ*m in diameter (Figure 1(a); Figure S2, Supplementary Materials). A significant increase was found in the adipocyte area of Non-Obese Lipedema participants (*p*=0.005) compared to Non-Obese Controls; there was no difference between the Obese groups (Figure 1(b)). The average adipocyte area was larger in Obese Controls compared to Non-Obese Controls (Figure 1(b)).

### 3.3. Immune Cells

**Figure 2:**
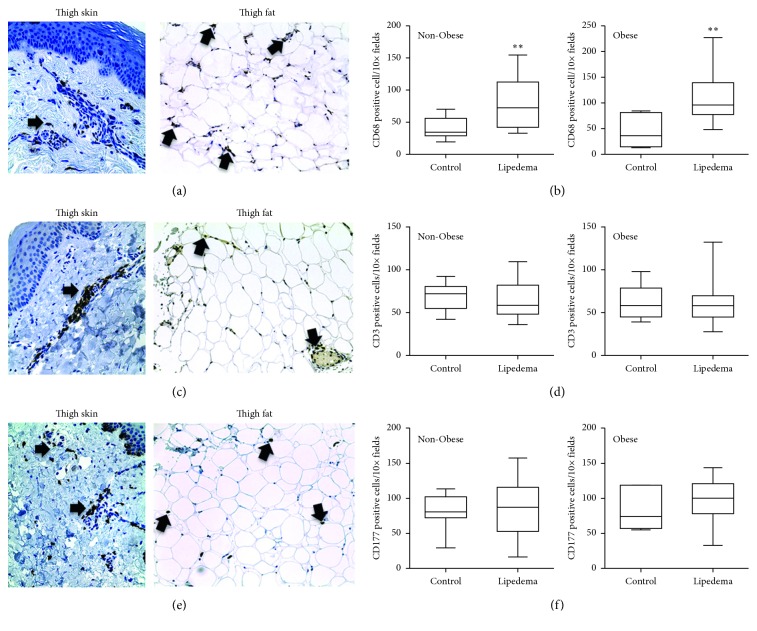
Immunohistochemistry of (a) CD68-labeled macrophages, (c) CD3-labeled T-lymphocytes, and (e) CD117-labeled mast cells in leg fat of lipedema participants (arrows). Images captured with 20x objective lens. Box and whisker plots of (b) CD68, (d) CD3, and (f) CD117 cell counts in skin and fat of control and lipedema participants. Non-Obese group (Control *n*=10; Lipedema *n*=10); Obese group (Control *n*=7; Lipedema *n*=12) (^*∗∗*^ *p* < 0.01).

The number of macrophages, but not T-lymphocytes or mast cells, was significantly higher in skin and fat of Non-Obese and Obese Lipedema participants compared to Controls in both groups (*p* < 0.05; Figures 2(b), 2(d), and 2(f)). Macrophages were detected throughout the skin in lipedema participants (Figure 2(a)) and around adipocytes in the hypodermal fat; in a few cases (12.5% in the Non-Obese Lipedema group and 14% in the Obese Lipedema group), macrophages encircled adipocytes forming crown-like structures (CLS). No CLS were observed in control groups. Mast cells and T lymphocytes were found primarily around blood vessels in the papillary dermal layer of the skin (Figures 2(c) and 2(e)) and around vessels in the fat in all participants.

### 3.4. Blood Microvessel Evaluation in the Papillary Dermis

**Figure 3:**
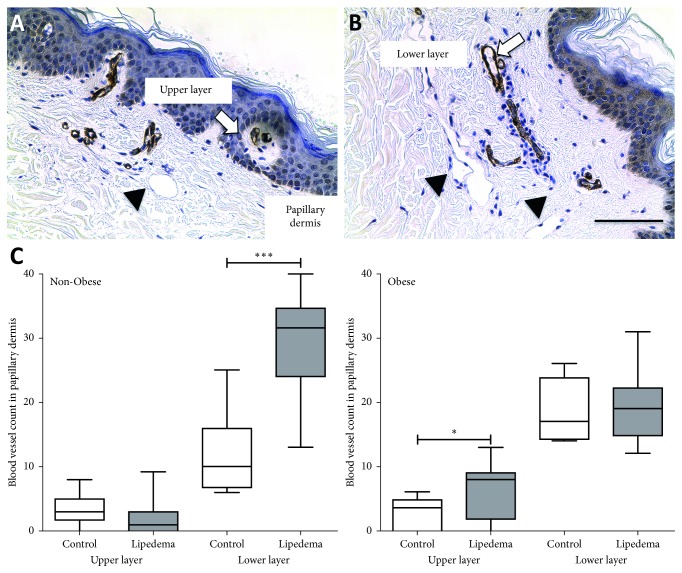
Number of blood vessels counted in the thigh skin of Non-Obese and Obese participants with and without lipedema (controls). (A, B) Immunohistochemistry of smooth muscle actin (SMA) positive vessels in the papillary dermal layer of lipedema leg skin (arrows). Note large lymphatic vessels in B (arrowheads) (scale bar 100 *µ*m). (C) Box and whisker plots representing the blood vessel count in groups. Non-Obese group (Control *n*=10; Lipedema *n*=13); Obese group (Control *n*=8, Lipedema *n*=15). (^*∗*^ *p* < 0.05, ^*∗∗∗∗*^ *p* < 0.001).

SMA staining revealed dermal blood vessels extending to the epidermis in lipedema participants and controls (Figures 3(a) and 3(b)). Quantification of these blood vessels showed a significant increase in their number in the lower papillary dermal layer in the skin in Non-Obese Lipedema participants compared to controls (*p* < 0.001; Figure 3(C)). The increase in blood vessel numbers positively correlated with macrophage numbers in the Non-Obese Lipedema group (Figures S3, Supplementary Materials, *p*=0.05, *r* ^2^=0.45) but not in the Obese group.

### 3.5. Blood Microvessel Evaluation in Fat

**Figure 4:**
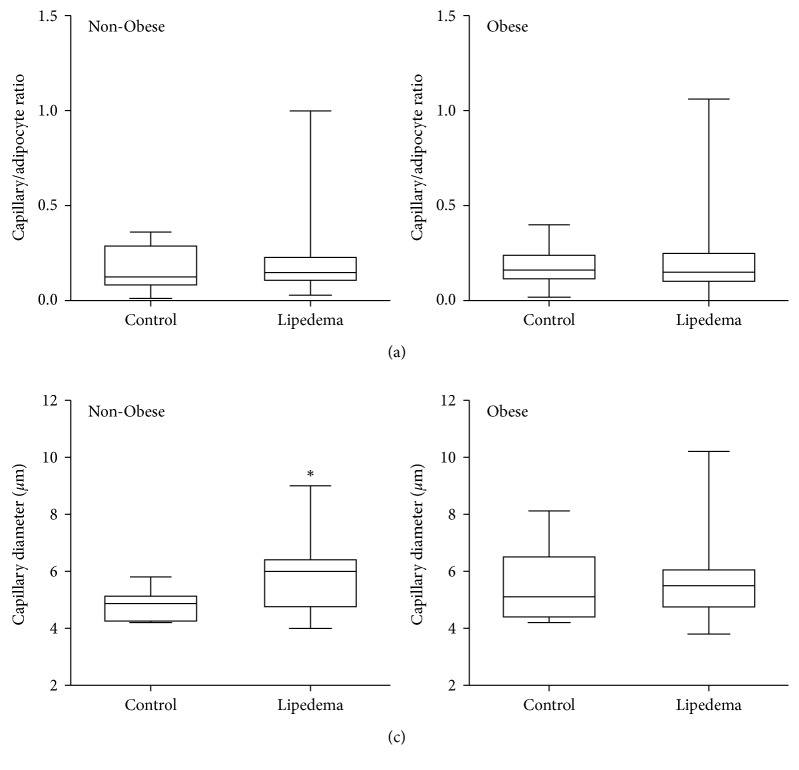
Quantification of capillaries in the leg fat of Non-Obese and Obese participants with and without lipedema (controls). Box and whisker plots of the (a) number of capillaries per adipocyte and (b) capillary diameter measured in Obese and Non-Obese Lipedema participants compared to Controls. Non-Obese groups (Control *n*=10; Lipedema *n*=13); Obese groups (Control *n*=8; Lipedema *n*=14).

The ratio of the number of capillaries to adipocytes in lipedema participants was similar to controls in both groups (Figure 4(a)). Capillary diameter was significantly larger in the fat of Non-Obese Lipedema participants but not in the Obese group, compared to controls (*p* < 0.05; Figure 4(b)).

**Figure 5:**
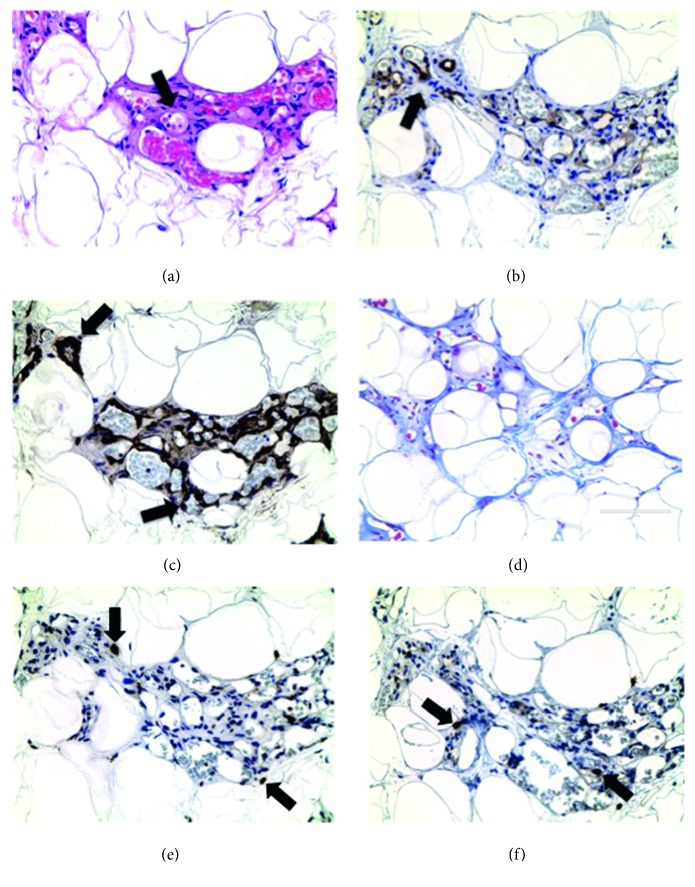
Angiogenesis in Non-Obese Lipedema leg fat. (a) H&E stain. Arrows indicate blood vessel lumens around adipocytes. (b, c) Immunohistochemistry of CD31 and SMA confirms the blood vessel origin of the tissue structures. (d) Trichrome stain demonstrating fibrosis in the areas of increased blood vessels. (e, f) Immunohistochemistry of CD68-labeled macrophages and CD117-labeled mast cells (arrows). Images captured at 40x objective lens (scale bar: 100 *μ*m).

Thirty percent of Non-Obese Lipedema participants developed a striking hypervascularity in the fat confirmed by CD31 and SMA positive stain (Figures 5(b) and 5(c)). Macrophages, mast cells, and fibrosis were detected in hypervascular areas mainly around blood vessels (Figures 5(d)–5(f)).

### 3.6. Lymphatic Microvessel Evaluation in the Dermal Skin

**Figure 6:**
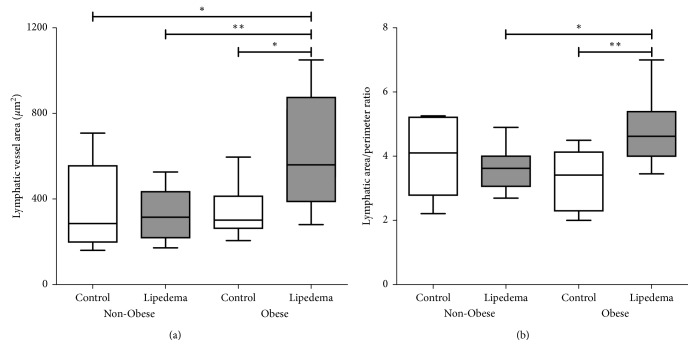
Measurement of the lymphatic vessel area and perimeter in the dermal skin of Non-Obese and Obese participants with and without lipedema (controls). (a, b) Box and whisker plots representing the quantification of lymphatic vessel area and area/perimeter ratio measurements. Non-Obese groups (Control *n*=7; Lipedema *n*=13); Obese groups (Control *n*=6; Lipedema *n*=14) (^*∗*^ *p* < 0.05; ^*∗∗*^ *p* < 0.01).

There was no significant difference in the number of lymphatic vessels between controls and lipedema in both groups. Examination of the morphological aspects of the vessels revealed a significant increase in the lymphatic vessel area in Obese Lipedema participants compared to Non-Obese Lipedema participants and Obese Controls (*p* < 0.01; Figure 6(a)). No significant difference was found for the lymphatic vessel perimeter within Obese and Non-Obese groups (Control and Lipedema). The aspect ratio of vessels from Obese participants tended to be higher than Non-Obese participants, but there was no significant difference between Control and Lipedema within the groups. The area/perimeter ratio (a measure of vessel stretch) was significantly higher in Obese Lipedema participants than either Control Obese or Non-Obese Lipedema participants (Figure 6(b), *p* < 0.01).

## 4. Discussion

Lipedema tissue case reports suggest lipedema fat has hypertrophic adipocytes [15]. In obesity, hypertrophic adipocytes are markers of inflammation, fibrosis, angiogenesis, and metabolically changed fat [14, 16]. This study was conducted to contrast lipedema fat to non-lipedema fat (controls) in groups of women, Non-Obese and Obese, to confirm or not previous case report data. The cellular architecture of adipose tissue, types and numbers of immune cells, and blood and lymphatic vessels, were investigated in an area where lipedema fat is prominent, the thigh.

Histological examination of fat demonstrated a heterogeneity in cell size in Non-Obese and Obese fat from lipedema participants as confirmed previously [15]; however, it was also present in controls; therefore, heterogeneity of adipocyte size is not a good marker of lipedema fat. The number of hypertrophic fat cells was greater in Obese compared to Non-Obese Controls as expected. However, in lipedema, the number of hypertrophic fat cells in the Non-Obese group was similar to that in Obese Lipedema and Control groups, and both were comparable in number the Obese Control group. Therefore, adipocyte hypertrophy in lipedema participants occurs independent of obesity by the usual measure of BMI.

The increase in adipocyte size in Non-Obese Lipedema participants occurred in association with increased macrophages in skin and fat as can be found in presumed non-lipedema obesity [17, 18]. These data suggest that lipedema fat in the gynoid distribution, even in non-obese women, has similar histological features of typical obesity. Finding CLS in lipedema fat has been reported previously [15, 19]. CLS are also found in adipose tissue from obese individuals but is unusual in the fat of non-obese individuals, suggesting that in lipedema fat, whether obese or non-obese, markers of a metabolically poor tissue are present, including dead adipocytes. The presence of CLS is also not consistent with the low rate of diabetes found in this population [20]. The location of the lipedema fat in the gynoid area may reduce the overall metabolic effect on the body, in part by lower postprandial triglyceride levels [21].

Angiogenesis, defined as the sprouting of new blood vessels from preexisting functional vessels and capillaries, is a multistep process involving vasodilation, matrix degradation, and activation and migration of endothelial cells to form new lumens [22, 23]. The papillary dermal skin in Non-Obese Lipedema participants in the lower layer had an increase in blood vessel numbers compared to controls, and Obese Lipedema participants had higher numbers of vessels in the upper layer of the papillary dermis suggesting increased angiogenesis in lipedema skin. The extension of the dermal vessels to the epidermal layer is a very interesting finding in lipedema as it has only been reported in psoriaform dermatoses [24, 25], inflammatory conditions of the skin.

While overall blood vessel count was not increased in lipedema fat by the capillary/adipocyte ratio, there was a significant dilation of capillaries in Non-Obese Lipedema participants as compared to Non-Obese Controls. Adding to that, angiogenesis was observed in the fat layer of 30% of Non-Obese Lipedema participants as demonstrated by abundant dilated capillaries surrounding adipocytes along with fibrosis and increased number of infiltrating immune cells.

This increase in blood vessel number and size in lipedema associated with an increase in macrophage numbers highlights an essential role of macrophages in inflammation and angiogenesis [16, 26, 27]. Increased blood vessels in the skin may therefore be an extension of underlying angiogenesis in fat and may be a marker of angiogenesis in lipedema.

Vascular endothelial growth factor (VEGF), a marker of angiogenesis, was previously reported to have elevated levels in the blood of ten women with lipedema compared to controls [28], consistent with these data. It is unclear why the robust angiogenesis occurred in the fat of some women with lipedema and not others but may relate to hypoxia in the tissue as suggested by Fife et al. [29]. The hypoxia may exist due to enlarged adipocyte cell size or excess fluid in tissue from dysfunctional microvasculature or venous insufficiency, both which need to be studied further in lipedema.

Changes in lymphatic vessels in skin from lipedema participants were subtler than in the blood vasculature and did not match the increased numbers of blood vessels found in the skin and fat. The increase in the size (area) and area/perimeter ratio of the lymphatic vessels in Obese Lipedema tissues fits well with other data showing that, even in clinically pure lipedema (uncomplicated by lymphedema), there is an expansion of the vessels but no significant changes in transport [30]. This may be an early compensatory event to increased lymph generation or downstream alterations in pressure in Obese Lipedema patients that is absent in Non-Obese Lipedema participants. The fact that most changes found herein in lymphatic vessel morphology were only in Obese Lipedema participants suggests that these individuals may have a greater risk of progressing to lipolymphedema than non-obese participants, consistent with clinical data [21, 31, 32].

Impaired dilation of capillaries is found in obesity [33], much different from the dense clusters of dilated capillaries found here in lipedema fat tissue. These capillary clusters appear similar to angiolipomas [8]. The increase in dilated capillaries and in lymphatic vessels in the skin (in Obese Lipedema participants) suggests an underlying cause for the occurrence of lipedema tissue, specifically alterations in capillary structure resulting in dilation and leakage of excess fluid that is not readily picked up by lymphatic vessels. The probable reason lymphatic size change is seen in Obese Lipedema and not in Non-Obese Lipedema is due to changes in peripheral perfusion resistance. Unlike the blood vascular system in which capillary size is dictated by a number of different factors (metabolism and local tissue signals), lymphatic vascular size is predominantly regulated by tissue fluid flux. In Non-Obese Lipedema patients, there is capillary dilation, which despite increased perfusion would not necessarily indicate a pathologically increased pressure, and therefore, no significant changes in the flux of fluid and solute from the capillary into the interstitial space and subsequently into the lymphatic are detected. The constricted capillaries (relative to Non-Obese Lipedema) of the Obese Lipedema patients would lead to the classically observed increased peripheral resistance in obesity, which given an equal central pressure and reflection coefficient of the capillaries, leads to increased blood vascular leak and increased lymph formation, and that is why lymphatic size increase is likely seen in Obese Lipedema and not in Non-Obese Lipedema [34, 35]. This excess fluid in the interstitial tissue may induce growth of lipedema fat [36] as well as hypoxia resulting in adipocyte cell death and the recruitment of macrophages. Fibrosis follows inflammation resulting in fat that is difficult to lose by extreme measures (overexercise, caloric-restricting or ketosis-generating diets, fasting, and bariatric surgeries) [37, 38]. These microvessel changes are especially intriguing considering that hypermobile joints have been found in a high percentage of women with lipedema, suggesting a connective tissue disorder [20]. The connective tissue alteration may promote changes in capillary structure signaling angiogenesis that is not mirrored by lymphangiogenesis, resulting in overload of the lymphatic system and fluid collection in the tissue, the definition of lipedema. Blood vessels are highly innervated. The dilated capillaries may alter sensory nerve function especially if accompanied by endothelial damage, and an inflammatory fluid-overloaded interstitial matrix within which nerves reside, might explain the pain experienced by lipedema patients; women with lipedema, especially those with Stage 3 or any lipolymphedema should be examined for neuropathy [39]. Preventing progression of the disease should include modalities that improve fluid flow through the tissue, protect the integrity of blood vessel structure, and reduce metabolic dysfunction of fat tissue.

## 5. Conclusion

Lipedema fat tissue even in non-obese women has classic features of obesity including hypertrophic fat cells, CLS, and increased macrophages. Dilated capillaries and angiogenesis in the lipedema tissue in the absence of concomitant lymphangiogenesis suggest an inherent structural defect in the capillaries allowing for increased flow of blood plasma into fat tissues and an inability to remove it. The excess fluid in the tissue may stimulate fat to grow, and the inflammation generated stimulates fibrosis and difficulty in weight loss. Inflammation and an interstitial fluid stagnation may activate nerve fibers resulting in the painful lipedema fat tissue. Strategies are needed to improve all aspects of tissue structure and flow.

## Figures and Tables

**Table 1:** Characteristics of control and lipedema subjects.

Characteristics	Control	Lipedema
*Non-Obese*		
*N*	10	16
Sex	Female	Female
Age	40.6 ± 4.2	43.3 ± 2.5
BMI	23.5 ± 0.7	25.2 ± 0.5
Stage 1	—	8
Stage 2	—	7
Stage 3	—	1
Diabetes	0	0

*Obese*		
*N*	9	15
Sex	Female	Female
Age	43.3 ± 5.2	46.1 ± 2.4
BMI	34.1 ± 1.4	34.1 ± 0.8
Stage 1	—	1
Stage 2	—	12
Stage 3	—	2
Diabetes	0	0

## Data Availability

The data used to support the findings of this study are included within the article and in the supplementary information file.

## Supplementary Materials

Supplementary Materials Supplementary Figure 1: methodology of lymphatic vessel measurements. Immunofluorescent images of Lyve-1 staining in human biopsy samples from control and lipedema participants. Images acquired at 20X with 2X digital zoom. Supplementary Figure 2: adipocyte size in Non-Obese and Obese participants with and without lipedema (controls). Box and whisker plot representing the average adipocyte size in Non-Obese (Control *n*=10; Lipedema *n*=10) and Obese groups (Control *n*=9, Lipedema *n*=11). Significance between the four groups was determined by one-way ANOVA followed by Tukey's post hoc test (^*∗∗∗*^ *p* < 0.001). Supplementary Figure 3: correlation between the number of blood vessels and CD68 + positive cells in Non-Obese Lipedema participants (*p*=0.05, *r* ^2^=0.44).
