# Supplementary material for: Dilated Blood and Lymphatic Microvessels, Angiogenesis, Increased Macrophages, and Adipocyte Hypertrophy in Lipedema Thigh Skin and Fat Tissue
Source: J Obes. 2019 Mar 3;2019:8747461. doi: 10.1155/2019/8747461 (PMC6425411; doi:10.1155/2019/8747461)
Supplement: Supplementary Materials — Supplementary Figure 1: methodology of lymphatic vessel measurements. Immunofluorescent images of Lyve-1 staining in human biopsy samples from control and lipedema participants. Images acquired at 20X with 2X digital zoom. Supplementary Figure 2: adipocyte size in Non-Obese and Obese participants with and without lipedema (controls). Box and whisker plot representing the average adipocyte size in Non-Obese (Control n=10; Lipedema n=10) and Obese groups (Control n=9, Lipedema n=11). Significance between the four groups was determined by one-way ANOVA followed by Tukey's post hoc test (∗∗∗ p < 0.001). Supplementary Figure 3: correlation between the number of blood vessels and CD68 + positive cells in Non-Obese Lipedema participants (p=0.05, r 2=0.44). [file 8747461.f1.pdf]

Supplementary Materials

Methodology of lymphatic vessel count

Area, perimeter and the aspect ratio of all vessels were measured and averaged for each sample using ImageJ software. Aspect ratio is the longest axis/shortest axis through the calculated center point of the vessel (Figure S1).

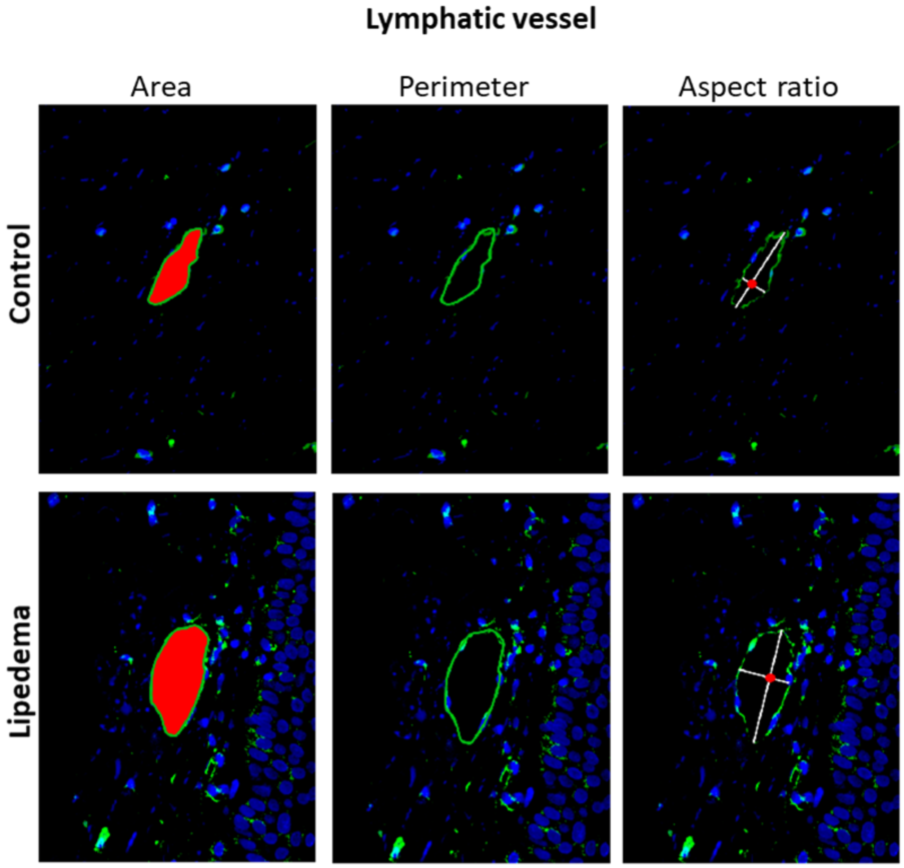

Supplementary Figure 1: Methodology of lymphatic vessel measurements. Immunofluorescent images of Lyve-1 staining in human biopsy samples from control and lipedema participants. Images acquired at 20X with 2X digital zoom.

Adipocyte size

A significant increase in adipocyte size was found in Non-Obese Lipedema compared to Non-Obese Controls (Figure S2). The hypertrophic adipocyte size in Non-Obese participants ranges from greater than 100 to ~ 150μm in diameter in Control and from 100 to ~170 μm in Lipedema. In Obese participants, adipocyte size ranges from greater than 100 to ~ 200μm in diameter in both Controls and Lipedema as shown in Figure S2.

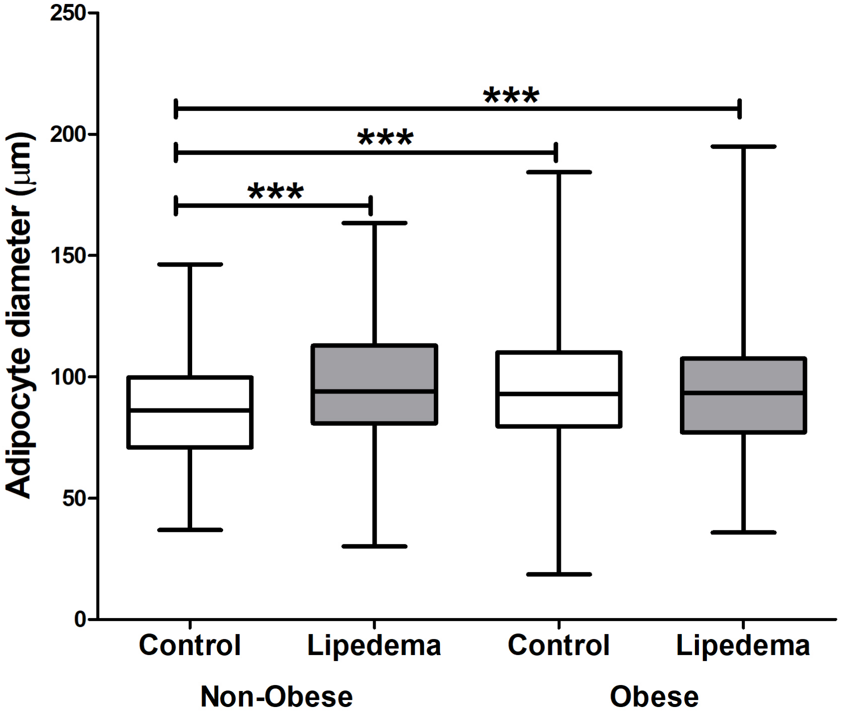

Supplementary Figure 2: Adipocyte size in Non-Obese and Obese participants with and without lipedema (controls). Box and whisker plot representing the average adipocyte size in Non-Obese (Control n=10; Lipedema n=10) and Obese groups (Control n=9, Lipedema n=11). Significance between the four groups was determined by One-way ANOVA followed by Tukey's post-hoc test. ***p<0.001.

Correlation between number of blood vessels and CD68$^+$ positive cells in Non-Obese Lipedema participants

The increase in blood vessel numbers positively correlated with macrophage numbers in the Non-Obese Lipedema group using Pearson method (Figures S3).

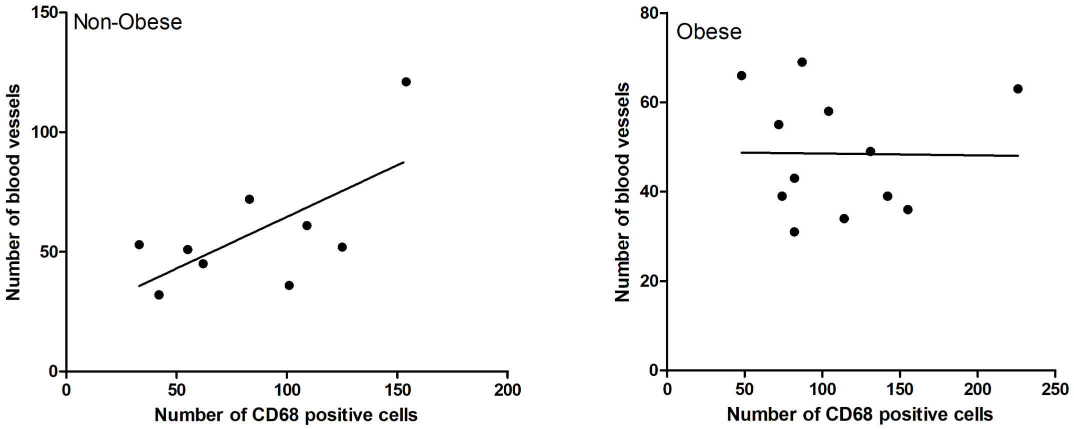

Supplementary Figure 3: Correlation between number of blood vessels and CD68$^+$ positive cells in Non-Obese Lipedema participants. p= 0.05, r^2=0.44.
